# Prognostic and Diagnostic Value of Spontaneous Tumor-Related Antibodies

**DOI:** 10.1155/2010/721531

**Published:** 2010-12-23

**Authors:** Sebastian Kobold, Tim Luetkens, Yanran Cao, Carsten Bokemeyer, Djordje Atanackovic

**Affiliations:** Department of Oncology, Hematology, Stem Cell Transplantation with the section Pneumology, University Cancer Center Hamburg (Hubertus Wald Tumorzentrum), University Medical Center Hamburg-Eppendorf, 20246 Hamburg, Germany

## Abstract

There is an urgent need for earlier diagnosis of malignancies and more stringent monitoring of relapses after antitumor therapy. In addition, new prognostic markers are needed for risk stratification and design of individualized cancer therapies. New diagnostic and prognostic parameters should overcome the impairments of current standards in a cost-effective manner. Serological approaches measuring spontaneous antibody responses against tumor-associated antigens could be of use as diagnostic and prognostic markers and could also be employed to evaluate response to therapy in cancer patients. Autoantibodies have been suggested to be of frequent and specific occurrence in patients with malignancies and to correlate with clinical parameters. Screening the relevant literature on this topic, we suggest that the analysis of single antibody specificities is unlikely to provide sufficient diagnostic and prognostic accuracy. The combined analysis of autoantibodies targeting different antigens, however, may reach high sensitivity and specificity. In addition, screening cancer patients for autoantibodies might identify subgroups with high relapse risk and a worse prognosis. Larger prospective trials should be initiated to identify sets of tumor-associated autoantibodies suited for the use in diagnostic algorithms for cancer detection and followup.

## 1. Introduction

For close to 150 years, human malignancies and the immune system have been suspected to be interaction partners [1]. While data supporting this relationship has accumulated in recent years, the exact biological role of spontaneously occurring anti-tumor immune responses is still a matter of controversy [2, 3]. In any case, the characterization of the crosstalk between tumors and their immune environment has led to a systematic analysis of the antibody repertoire of cancer patients [4]. The relatively high frequency of spontaneous antibody responses against cancer-related antigens led to the assumption that these antibodies could be of use in the clinical setting [5]. Accordingly, a lot of effort was invested in correlating the presence of such antibodies with clinical parameters to assess their use as prognostic parameters. Furthermore, the highly cancer-specific nature of some of these antibodies resulted in the evaluation of their diagnostic utility [6]. Both approaches seemed very promising as a serological detection of cancer, and a serologic risk stratification would be easy to handle, of low cost, and much more likely to be accepted by a wide majority of patients hesitant to undergo invasive procedures [7].

Nevertheless, the initial euphoria was dampened by controversial results regarding the prognostic reliability of tumor-associated autoantibodies throughout different cancers [8]. Autoantibodies were either reported to improve the prognosis of cancer patients, to worsen the clinical outcome, or even to be irrelevant for the course of the disease [9]. From a diagnostic point of view, the results did not meet the high expectations perhaps as the analysis of single autoantibodies proved to be of insufficient sensitivity for clinical routine [8].

Very recently, the idea that tumor-associated autoantibodies could be developed into meaningful diagnostic and prognostic tools has been revived [10, 11] as researchers aimed at increasing the sensitivity of serological assays by combining several autoantibodies [12]. In the present paper, we will try to answer the question whether and how autoantibodies could be used to enhance early diagnosis of malignant conditions and how they might contribute to perform appropriate risk stratifications in these patients.

## 2. Serological Analyses in Cancer Patients

### 2.1. Tumor-Associated Autoantibodies against Single Antigens Lack Sensitivity to Reach Diagnostic Relevance

Since tumor-associated autologous antibodies have first been observed, it has been investigated whether they could be used as an early disease marker in a minimally invasive diagnostic approach [6]. In order to be applicable as diagnostic markers, tumor-associated autoantibodies should only be present in cancer patients, they should be detectable in as many patients as possible, and they should ideally appear early in the course of the disease.

Choosing an appropriate antigen is a difficult task in light of the overwhelming amount of antigens eliciting autoantibodies in cancer patients. The Cancer Immunome Database [13] currently lists 2,743 sequences for 2,316 clones, and this number is constantly growing. However, most antigens are unsuitable for diagnostic purposes because they are too low-titered, occur only in a subgroup of cancer patients, and/or are also found in healthy subjects or patients with benign diseases [14].

We screened all available studies evaluating autoantibodies as possible diagnostic parameters in cancer patients in the pubmed database. Autoantibodies had to be investigated in at least five studies in order to be included into the final analysis. We proposed three quality criteria for the 9 antibody specificities which were analyzed for their diagnostic value. At least two of these criteria had to be fulfilled by a certain antibody in order to qualify as a promising candidate for diagnostic purposes. Serological responses (1) had to be high-titered (defined as a titer above 1:1,000), (2) had to be detectable in at least 40% of patients with a respective malignancy, and (3) had to be absent from the peripheral blood of healthy subjects. Only three types of antibodies fulfilled our quality criteria: polymorphic epithelial mucin (MUC-1), p53, and Sry-like high mobility group super family (SOX).

MUC-1 is overexpressed by breast, colorectal, and lung cancer and is deficiently glycosylated on the cell surface [15]. As a surface antigen, the immunogenicity of MUC-1 was of particular interest and has been extensively studied [16, 17]. Despite the fact that high-titered MUC-1-specific autoantibodies are frequently found in cancer patients, their use as single diagnostic parameters is hampered by the circumstance that they can be detected at an almost similar frequency in healthy donors [18].

In contrast, in a study examining patients with lung cancer, p53-specific autoantibodies could be observed years prior to detection of malignant lesions [8]. All patients developed cancer after first detection of anti-p53 autoantibodies and the serological response remained detectable throughout the remaining course of the disease [8] In light of such data, p53-specific autoantibodies would seem to be very well suited for diagnostic purposes [19, 20] and, accordingly, anti-p53 autoantibodies have been extensively studied regarding their diagnostic and prognostic applicability for a wide variety of malignancies [21–23]. Unfortunately, one conclusion from these investigations is that screening for anti-p53 antibodies—at least when used as a single antigen—is of insufficient sensitivity, which is indicated by a maximal cancer detection rate of 46% [24]. In addition, p53 antibodies are not restricted to a single cancer type [23] and the observation that anti-p53 antibodies are detectable in subgroups of patients with inflammatory diseases who never develop a malignancy represents another important downside of the use of p53 serology for diagnostic purposes.

Antibodies against SOX families B1 and B2 are found [25] in up to 43% of patients with small cell lung cancer (SCLC) [10]. Unfortunately, the presence of SOX-specific antibodies in patients with benign diseases [10, 26] results in an insufficient specificity of SOX when used as a single antibody. Anti-SOX antibodies have recently been suggested to help differentiating between patients with paraneoplastic (cancer associated) and sporadic Lambert-Eaton syndrome, respectively [10]. On the other hand, many tumor patients will evidence SOX antibodies without any hint for neurological symptoms [25]. Additional well-designed studies are needed before the question whether anti-SOX antibodies are usable for diagnostic purposes can be resolved. 

A very recent meta-analysis [27] has concluded that EBV-specific antibodies are of diagnostic value for the diagnosis of nasopharyngeal carcinoma. Specificity and sensitivity were suggested to be extremely high for a single antigen (over 90%). However, these retrospective analyses still need to be validated in a prospective manner. At this time, one has to conclude that antibodies with a single specificity cannot reliably be used for cancer screening and early detection.

### 2.2. The Combination of Tumor Antigen-Specific Autoantibodies May Reach Diagnostic Relevance

As a single cancer type potentially elicits antibody responses against a large number of antigens, another approach is to combine several types of antibody responses in a diagnostic algorithm. Such a strategy should allow for increased sensitivity without significantly complicating the diagnostic procedure. However, as of yet only a small number of studies has addressed this issue. We will herein only discuss combinations of antibody specificities (1) showing a specificity of at least 75% being investigated in studies (2) including at least 100 patients. Most published studies did not fulfill these criteria (Table 1). Most of these studies used samples of patients in whom the diagnosis of cancer had already been established.

It has recently been shown that up to 75% of all breast cancer patients bear antibodies against at least one of the following antigens: p53, c-myc, NY-ESO-1, BRCA1, BRCA2, HER2, or MUC1. With a combined analysis of these antibodies, specificity for breast cancer increased to 85% while sensitivity remained relatively low with 64% [17]. On the other hand, the combined analysis of antibodies to p53, HER-2 (Human Epidermal growth factor Receptor-2), IGFBP-2 (Insulin like Growth Factor Binding Protein 2), and TOPO2*α* (Topoisomerase-2-alpha) increased both diagnostic specificity and sensitivity to up to 75% for breast cancer patients [12].

Combined serological approaches have also been applied to other malignancies. Using lysates of a human pancreatic carcinoma cell line as a mixture of unknown target antigens, antibody responses were detected in over 90% of patients with pancreatic cancer with a specificity of 80% [39, 42]. In another study 22 phage-displayed tumor-associated antigens were combined to diagnose prostate cancer in a serological analysis. Sensitivity of this approach was remarkably high with 88% while still detecting 82% of true-positive patients [40]. Very recently, a large study including over 600 patients with lung cancer and utilizing a combination of five antigens reached a remarkable 90% specificity, while sensitivity remained relatively low with 40% [41].

In conclusion, the combined analysis of different tumor-related autoantibodies might represent a promising approach for the diagnosis of cancer leading to an up to threefold increase in specificity and sensitivity (Table 1). However, despite the general scalability of this approach, increasing costs and the methodological complexicity of large antibody screens become important considerations. To be applicable in the clinical setting, standardized and reliable methods for the performance of serological concepts are needed. Unfortunately, a large variety of different methods for antibody detection has been used worldwide and results from these analyses are often difficult to compare [43]. Recently, a semiautomatic ELISA assay has been proposed to possess high specificity, reproducibility, and reliability [41]. The broad introduction of comparable methods probably represents a prerequisite for the future use of serological analyses of antibodies in clinical routine. Comprehensive prospective studies are needed to provide a definitive rationale for their application in the clinical routine.

### 2.3. Tumor-Related Autoantibodies Are Predictors of Clinical Outcome in Cancer Patients

In spite of significant progress in the diagnosis and followup of cancer patients, it is still difficult to stratify patients according to their risk of relapse [11]. Such an assessment would be of great use to both patient and clinician, as it would help to differentiate between those who might benefit from additional therapy and those who may only suffer from therapy-related side effects without achieving an improved outcome. An ideal stratification technique would also be of low cost and cause minimal discomfort for the patient. Obviously, a serological approach can fulfill both of these criteria and add further diagnostic aspects not covered by current stratifications. Many studies have examined the correlation between the occurrence of tumor-related autoantibodies and the clinical outcome of cancer patients (Table 2). When we screened the literature, we focused on three types of target antigens: (1) shared cancer-specific antigens, (2) antigens overexpressed in tumors compared to normal tissues, and (3) mutated antigens. Overall, among the studies dealing with the prognostic impact of autoantibodies in cancer patients, 50% were unable to demonstrate an impact on the course of the disease, 33.33% did show a worse outcome, and 16.67% involved a better prognosis.

A large body of studies particularly involving SEREX (Serological Identification of antigens by recombinant expression cloning) identified antibodies that did not reveal any significant impact on clinical outcome. Even antibodies against antigens that are involved in the pathogenesis of the disease (Cyclin B1 or p53 in oral cancer) or associated with a more aggressive course did not seem to affect the outcome (Table 2). 

Antigen p53 probably represents the tumor-associated protein that has most extensively been studied with regard to its prognostic value. Several small studies yielded variable results, ranging between a missing effect and a negative influence on the patients' outcome [71]. Interestingly, one larger study performed in hepatocellular carcinoma patients suggested that the presence of p53-specific antibodies might be associated with an increased overall survival [72]. However, a number of other large studies in breast, lung, colon and oral cancer patients as well as a meta-analysis clearly highlighted the correlation between the presence of p53-specific autoantibodies and decreased overall and progression-free survival [19, 73].

Another well-studied example of antibody responses associated with a poor prognosis is found among Cancer Testis Antigens (CTA). CTA, also called shared cancer-specific antigens, represent a unique class of tumor proteins with an expression restricted to normal testis and cancer tissue. Interestingly, antibodies against some CTA, such as NY-ESO-1, were primarily found in patients with advanced disease [74]. Levels of NY-ESO-1-specific antibody responses seemed to correlate with the volume of tumor tissue present in the patient's body [67]. Accordingly, spontaneous occurrence of NY-ESO-1 antibodies has particularly been found in multiple myeloma patients and melanoma patients who relapsed and/or suffered from progressive disease [67, 75]. Moreover, in prostate cancer, the presence of anti-NY-ESO-1 antibodies was associated with a decreased overall survival while no influence of NY-ESO-1 seropositivity was observed in patients with esophageal cancer [47, 68]. These findings might, on the one hand, suggest that NY-ESO-1-specific antibodies merely represent markers of an increased tumor load and do not indicate a biologically relevant immune response. Conversely, NY-ESO-1-specific antibodies might in fact exert a relevant anti-tumor effect by directly and/or indirectly suppressing tumor growth in earlier stages of the disease. As the disease progresses, the net effects of these cancer-specific immune responses might decrease and, finally, they will be overwhelmed by cancer-related immunosuppression and the kinetics of tumor growth [67]. This hypothesis would, to some degree, be supported by observations in patients vaccinated with CTA where response to therapy and enhanced overall survival have been associated with CTA-specific T cell and antibody responses [76].

On the other hand, a number of studies have suggested a positive influence of the occurrence of tumor-associated autoantibodies on the outcome of the patient. In one study with NSCLC patients, the presence of anti-MUC-1 antibodies was clearly linked to earlier stages of the disease as well as a lower recurrence rate [15]. Based on these findings and on *in vitro *data, it was hypothesized that spontaneously occurring anti-MUC-1 antibodies might bind to the surface of tumor cells uncovering adhesion molecules masked by the deficiently glycosylated MUC-1. According to this hypothesis, this would then render MUC-1 specific antibodies a part of an effective immune response against cancer cells.

Compared to the overall number of studies analyzing autoantibody profiles in cancer patients, relatively few studies evaluate the impact of these immune responses on the patients' prognosis. This observation may be an expression of publication bias, as negative results are less likely to be reported than positive ones [77]. Accordingly, controversial results are found even for a given antigen [19]. In contrast, antigens such as p53 or MUC-1 are well studied in large cohorts and results may be considered reliable. Accordingly, even if most studies suggest for autoantibodies to have no impact on the course of human malignancies, confirmation of these results is strongly needed. Importantly, no study has yet revealed how such antibodies impact on the patient's prognosis, and it is unclear whether autoantibodies themselves actively contribute to the patient's outcome or whether they are only an indicator of the clinical condition.

As in the case of diagnostic approaches, one could theoretically analyze antibody responses against different antigens in a combined manner in order to enhance the prognostic impact of such assays. A very recent work by Gnjatic et al. has addressed this issue and found the simultaneous presence of four different autoantibodies to define a subgroup of patients who showed an improved prognosis [49]. This pioneering work might lead the way towards a more efficient prognostic classification of cancer patients based on serological analyses.

Unfortunately, to date little is known regarding the behavior of humoral responses against tumor antigens over the course of the disease. In the vaccination setting antibody responses have been routinely monitored over time, but longitudinal studies on spontaneously occurring antibody responses are scarce [78]. The few data available suggest that, for example, the appearance and persistence of NY-ESO-1-specific antibodies are tightly linked to the amount of antigen present in the patient's body. Accordingly, disease progression appears to promote the development of NY-ESO-1-specific antibody responses and to lead to increased antibody titers. Future studies will have to determine in a stringent manner the clinical conditions contributing to the development of autoantibodies in cancer patients. 

In any case, the biological basis of either of these observations remains elusive. Therefore, although it is impossible to design randomized studies in this particular setting, prospective analyses evaluating such antibody responses over the course of the patient's disease may help to address two related questions: (1) under which clinical situations does the patient develop antibodies against tumor-associated antigens and (2) what is the temporal association between clinical incidents (i.e., response to therapy or relapse) and the development of humoral immune responses.

## 3. Conclusions

The large diversity of the antibody repertoire directed against autologous tumor-related antigens illustrates the complex and heterogeneous nature of the human seromic repertoire. As summarized in this analysis, the combination of a few frequently occurring types of antibodies may be of diagnostic value for certain malignancies. The combination approach appears particularly attractive because of the lack of secondary prevention tools for many tumor types. However, prospective studies evaluating the true value of this method are required prior to clinical introduction.

As primary cancer prevention remains a frustrating issue due to the multifactorial origins of cancer, early detection of cancer lesions is an essential goal to enhance the patient's prognosis and to reduce costs. Current strategies focus on apparative diagnostics and only to a much lesser extend on serological testing [79, 80]. As of yet, only mammography, colonoscopy, cervical smear, and skin examination are accepted early cancer detection tools for the respective cancer types [79, 81–83]. While the reduction in overall mortality provided by these screening methods alone is not well defined, specificity and the sensitivity reach almost 90% in given studies [79]. In contrast, it is still not completely clear to what extent approaches, such as the measurement of prostate-specific antigen in the serum, improve the patient's outcome [80]. 

Currently, most prognostic markers used in a routine clinical setting are based on clinical parameters such as performance status and stage and vary between cancer types [84]. In addition, molecular markers such as the expression of HER2/NEU have been established to further stratify patients according to their relapse risk and their need for additional therapies [85]. It is very likely that, in the near future more sophisticated tools such as gene expression profiles will become available [86]. 

Two major hurdles still hamper the wide spread use of the above-mentioned early detection tools in cancer care: (1) the majority of patients fear invasive and time-consuming procedures and (2) costs of modern diagnostics. Accordingly, there is a need for minimal invasive tools which are cost-effective, and the analysis of cancer-specific antibodies might provide an acceptable solution to this clinical dilemma. Such a role would certainly be complementary as serological approaches are unlikely to replace “classical” pathological markers or staging procedures.

In our opinion, the available data justifies cautious optimism regarding the development of serological analyses into diagnostic and/or prognostic tools. Detailed analyses of the human antibody repertoire will hopefully contribute to clarifying the clinical value of such humoral responses. Combined data from these studies could vastly improve the identification and selection of appropriate diagnostic and prognostic markers as well as of future targets for immunotherapies [87, 88].

## Figures and Tables

**Table 1:** Combinations of autoantibodies evaluated as diagnostic measures.

Antibody specificity	Number of patients	Tumor type	Specificity/Sensitivitiy	Ref.
PIM1, MAPKAPK3, ACVR2B	114	Colon cancer	74/83	[28]
ASB-9, SERAC1, RELT	87	Breast cancer	100/80	[29]
PPIA, PRDX2, FKBP52, MUC-1, HSP60	142	Breast cancer	73/85	[30]
Calnuc, p53, Cyclin D1, Cyclin B1, myc	447	Mixed	59/62	[31]
myc, p53, Cyclin B1, p62, Koc, IMP1, Survivin	976	Mixed	50/91	[22]
CCCAP, HDAC5, p53, NMDAR, NY-CO-16	94	Colon cancer	59/78	[32]
SEREX clones (*N* = 6)	48	Colon cancer	96/92	[33]
SEREX clones (*N* = 10)	77	HCC	87/66	[34]
Imp1, p62, Koc, p53, myc, Cyclin B1, Survivin, p16	142	HCC	50/60	[35]
SEREX clones (*N* = 80)	39	Head and neck cancer	90/80	[36]
O-glycopeptides	56	Mixed	85/25	[37]
Nucleophosmin, Cathepsin D, p53, SSX	125	Ovarian cancer	NN	[38]
Autoantibodies to MIAPACA cell line	238	Pancreatic cancer	80/93	[39]
Phage display clones (*N* = 22)	119	Prostate cancer	88/82	[40]
p53, c-myc, HER2, NY-ESO-1, BRCA1, BRCA2, MUC1	97	Breast cancer	85/64	[17]
p53, NY-ESO-1, CAGE; GBU4-5, Annexin 1	626	Lung cancer	90/40	[41]

**Table 2:** Influence of autoantibodies against tumor-associated antigens on the prognosis of cancer patients.

Autoantibody specificity	Number of patients	Tumor type	Impact	Ref.
Laminin	71	Breast cancer	Decreased OS	[44]
HSP90	327	Breast cancer	Decreased OS	[45]
p53	9489	Breast, gastric, colon cancer, NSCLC and oral cancer	Decreased OS	[46]
NY-ESO-1	207	Prostate cancer	Decreased OS	[47]
Nucleophosmin	100	Breast cancer	Decreased RFS	[48]
Panel of 29 antigens	60/59	Ovarian cancer/pancreatic cancer	Increased OS	[49]
CTSP-1	147	Prostate cancer	Increased RFS	[50]
p53	130	HCC	Increased RFS	[51]
Laminin-Receptor	67	CLL	Increased RFS	[52]
CML66	15	CML	Increased RFS	[53]
GLEA3 and PHF3	62	Glioblastoma	Increased OS	[54]
MUC1	30	NSCLC	Increased RFS	[18]
MUC1	100	Ovarian cancer	Increased OS	[55]
MUC5AC	30	Colon cancer	Increased OS	[56]
SOX1	90	SCLC	Increased OS	[57]
CEA	52	Breast cancer	Increased RFS	[58]
SEREX clones	12	Meningioma	None	[59]
SCP1	100	Pancreatic cancer	None	[60]
Cyclin B1	42	AML	None	[61]
p53	120	Oral cancer	None	[6]
Hsp90	116	Ovarian cancer	None	[62]
ALK	21	ALL	None	[63]
SEREX clones	25	SCC	None	[64]
MUC-1	125	Breast cancer	None	[65]
Survivin	76	NSCLC	None	[66]
NY-ESO-1	12	Different cancers	None	[67]
NY-ESO-1	69	Esophageal cancer	None	[68]
Phage Display clones	176	Breast cancer	None	[69]
Braf	372	Melanoma	None	[70]

OS: overall survival, RFS: recurrence-free survival.
